# Shiitake mushroom powder supplementation increase antioxidative activity in dogs

**DOI:** 10.3389/fvets.2024.1355560

**Published:** 2024-06-19

**Authors:** Akio Kusaba, Toshiro Arai

**Affiliations:** School of Veterinary Medicine, Nippon Veterinary and Life Science University, Musashino, Japan

**Keywords:** antioxidative activity, dog, shiitake, sirtuin1, superoxide dismutase

## Abstract

**Introduction:**

The prevalence of age-related diseases, including obesity (a lipid metabolism disorder), increases with the increase in a dog’s lifespan. Most of age-related diseases are associated with oxidative stress by excessive production of reactive oxygen species (ROS) from impaired mitochondrial functions. Safe and effective supplements with antioxidative and anti-inflammatory activities are required to prevent obesity and associated complications. Shiitake mushroom exhibit various functions including antioxidant activity. We investigated the effect of shiitake powder supplementation in healthy dogs.

**Methods:**

Shiitake powder was supplemented at a dose of 800 mg/kg body weight/day for 4 weeks. The dose was set as 0.60–0.65 mg/kg/day of eritadenine, a hypocholesterolemic factor.

**Results:**

The body weight and body condition score of the dogs did not change after shiitake supplementation. In contrast, plasma total cholesterol concentrations decreased and superoxide dismutase activity and leukocyte sirtuin1 mRNA expression increased significantly in the dogs that received the supplement.

**Discussion:**

Oral administration of shiitake powder increased antioxidative activity. The supplement may be useful in ameliorating the signs of age-related diseases, including obesity, in dogs.

## 1 Introduction

As the life span of dogs has increased, the prevalence of age-related diseases has also increased (1, 2). In recent years, the concept of “inflammaging” has attracted considerable attention (3). The process refers to chronic low-grade inflammation in elderly tissues and is involved in the development of age-related diseases (4). Oxidative stress (OS) is associated with various age-related conditions, including sarcopenia and frailty (5, 6), and OS-induced aging and associated disorders cause deterioration in soft tissues and disrupt homeostasis (7, 8). Obesity, a lipid metabolism disorder, is categorized among age-related diseases (9) and is caused by an imbalance between energy consumption and expenditure that leads to ectopic lipid accumulation (10). Factors responsible for OS generation during obesity include hyperglycemia, elevated tissue lipid levels, vitamin and mineral deficiencies, chronic inflammation, hyperlipidemia, endothelial dysfunction, and impaired mitochondrial function. All these complications lead to excessive production of reactive oxygen species (ROS) and are intricately associated with obesity and associated complications, especially insulin resistance and type 2 diabetes mellitus (11).

The liver plays a central role in obesity-associated metabolic changes. Hepatic mitochondrial dysfunction can cause alterations in fat oxidation, ROS production, and OS (12). Amelioration of liver function is important for the prevention of obesity and related metabolic disorders (13, 14). Safe and effective foods and supplements with antioxidative and anti-inflammatory activities are required to prevent obesity and associated metabolic disorders with aging in dogs.

The shiitake mushroom, *Lentinus edodes*, is a basidiomycete that has been consumed for more than 2,000 years because of its nutritional value and health benefits (14). Shiitake mushrooms have low lipid content, high fiber content, and a considerable amount of protein, and their extracts and pure compounds exhibit antibacterial, antifungal, cytostatic, antioxidant, anticancer, and immunomodulatory activities (15, 16). Eritadenine, a hypocholesterolemic factor isolated from shiitake mushrooms, has been reported to suppresses the biosynthesis of cholesterol in the liver and decrease plasma cholesterol concentrations in rodents (16, 17). Shiitake mushrooms contain various components besides eritadenine to support good health and appear to be a useful food source for ameliorating hepatic functions in animals (16).

The objective of this study was to investigate the effects of shiitake powder supplementation on liver function and antioxidative activities in healthy dogs and to discuss the usefulness of shiitake powder as an anti-obesity supplement for dogs.

## 2 Materials and methods

### 2.1 Animals

Eight healthy Beagles [four males and four females; 1–3 years old; body weight (BW), 9.4–11.4 kg; body condition score (BCS), 4.0–6.0] were used in this study. All dogs were kept individually in cages measuring 54 cm (height) × 45 cm (wide) × 72 cm (depth) for 4 weeks under the same conditions at the Narita Animal Science Laboratory Co., Ltd. (Narita, Japan). The environment was maintained at 24.0 ± 2.0°C and 55.0 ± 10.0% relative humidity, and on a 12:12 h, light: dark cycle (light on 8:00 a.m. to 8:00 p.m.) The dogs were fed commercial diet (DS-A; Oriental Yeast Co. Ltd., Tokyo, Japan) at 8 am daily. The commercial plastic food bowls were used, and washed by hand and dried after each meal. Body weight and BCS were measured before (week 0) and 4 weeks after shiitake powder supplementation. Body condition scores were evaluated using a 9-point scale system, ranging from very thin (BCS = 1) to ideal (BCS = 5) and severe obesity (BCS = 9) (18). The animal study protocol was approved by the Narita Animal Science Laboratory Co., Ltd. Research Animal Ethical Committee (No. 21-C034).

### 2.2 Shiitake powder supplementation

Shiitake powder was prepared by Mori & Company, Ltd. (Kiryu, Japan). Fresh shiitake mushroom was frozen in the freezer for 24 h. It was then dried in a freeze dryer with a sublimator and vacuum station. The average operating pressure in the drying chamber reached 0.01–0.05 mbar. After dehydration process, the obtained dried mushroom was ground to fine powder using a grinder. One hundred grams of freeze-dried powdered shiitake contains 78 mg of eritadenine as hypocholesterolemic factor. Shiitake powder was supplemented at a dose of 800 mg/kg body weight/day for 4 weeks, corresponding to 0.60–0.65 mg/kg/day of eritadenine (19), a hypocholesterolemic factor in shitake mushroom. The control dogs (two females and two males) did not receive the supplement.

### 2.3 Blood sampling

Five milliliters of blood were collected from the jugular vein of each animal into heparinized tubes before (week 0) and 4 weeks after supplementation. Blood collection was performed before the morning feeding and the collected samples were immediately centrifuged at 2,000 × g for 10 min at 4°C to obtain plasma. The plasma samples were stored at −80°C until use.

### 2.4 Metabolite, hormone, and enzyme analyses

Plasma glucose, total cholesterol, triglyceride, and total protein concentrations, and aspartate aminotransferase (AST) and alanine aminotransferase (ALT) activities were measured using an autoanalyzer (JCA-BM2250; JEOL Ltd., Tokyo, Japan) with the manufacturer’s reagents at FUJIFILM Vet Systems (Tokyo, Japan). Free fatty acid (FFA) concentrations were measured using a commercial kit (NEFA-C test kit; Wako Pure Chemical Industries, Inc., Tokyo, Japan). Plasma insulin and adiponectin concentrations were measured using a rat insulin ELISA kit (AKRIN-010T; Shibayagi Co., Gunma, Japan) and a mouse/rat adiponectin ELISA kit (Otsuka Pharmaceutical Co., Ltd., Tokyo, Japan), respectively. Antibodies used for measurement of insulin and adiponectin were not specific to the canine species. The concentrations of insulin and adiponectin were relative values against the rat insulin and adiponectin antibodies, respectively. Superoxide dismutase (SOD) activity was measured using a commercial kit (SOD Activity Assay kit NWK-SOD02; Northwest Life Science Specialties, LLC, Vancouver, WA).

### 2.5 Quantitative real-time PCR analysis of sirtuin1 mRNA

Total leukocyte RNA was extracted from the blood samples using TRIzol (Invitrogen, CA), according to the manufacturer’s protocol. Total RNA (1 μg) was reverse transcribed using a QuantiTect Reverse Transcription Kit (Qiagen, Hilden, Germany). Genomic DNA was removed by DNase treatment and cDNA was synthesized. After inactivating the reverse transcription reaction by heating at 95°C for 3 min, the cDNA product was used for quantitative real-time PCR (q-PCR). Reactions were carried out with Perfect Real Time TYBR Premix Ex Taq II (Takara, Tokyo, Japan) on an ABI 7300 Real Time PCR Sequence Detection System (Applied Biosystems, Foster City, CA) and the following shuttle PCR protocol: 95°C for 30 s, followed by 40 cycles of 95°C for 5 s, and 60°C for 35 s, in 20 μL reaction volumes containing 2 μL template cDNA, 0.8 μL primers (final concentration: 0.4 μM, including 0.4 μL ROX Reference Dye), and 6.0 μL distilled water. The primers listed in Table 1 were used for sirtuin1 and β-actin mRNA quantitation (20). Following real-time PCR, the fragment was subjected to dissociation-curve analysis to avoid nonspecific PCR amplification. Each sirtuin1 mRNA value was normalized to that of β-actin.

**Table 1:** Primer sequences used in quantitative RT-PCR analysis of peripheral leucocyte mRNA from dogs.

	PCR product length (bp)	Primer type	Primer sequences (5′–3′)	Gene Bank accession number
SIRT1	145	Sense	CGCCTTGCAATAGACTTCCC	XM_546130
		Anti-sese	TGAATTTGTGACAGAGAGATGGTTG	
β-Actin	129	Sense	GCCAACCGTGAGAACATCACT	AF021873
		Anti-sense	CCCAGAGTCCATGACAATACCAG	

### 2.6 Statistical analysis

All measured values are expressed as means ± standard error (SE). Statistical significance was determined using a paired *t*-test. The significance level was set at *p* < 0.05. The statistical analyses were performed using Microsoft Excel.

## 3 Results

### 3.1 Body weight and body condition scores of dogs

None of the dogs showed any signs of disease during the experiment. Body weight and BCS of the dogs did not change after shiitake supplementation (Table 1).

### 3.2 Plasma metabolite and hormone concentrations and enzyme activities in dogs

In the plasma of the dogs that received the shiitake powder, total cholesterol concentrations decreased significantly and there was a tendency toward increase in FFA concentrations, but the increase was not significant. Plasma glucose, triglyceride, total protein, insulin, and adiponectin concentrations did not change after the shiitake supplementation. Plasma AST and ALT activities did not change after the shiitake powder supplementation (Table 1).

### 3.3 Molecules related to oxidative stress in dogs

Sirtuin1 mRNA expression in the leukocytes increased significantly in the dogs fed the shiitake powder-supplemented diet. Plasma SOD activities increased to five times with the shiitake powder supplementation (Table 2).

**Table 2:** Changes in body weight, body condition score, plasma metabolite and hormone concentrations, and enzyme activities in dogs fed diet supplemented with shiitake powder.

	Control (*n* = 4)		Shiitake supplemented (*n* = 4)	
	Week 0	Week 4	Week 0	Week 4
Body weight (kg)	10.6 ± 0.3	10.9 ± 0.4	10.4 ± 0.4	10.9 ± 0.5
Body condition score	5.0 ± 0.4	5.0 ± 0.4	5.0 ± 0.4	5.0 ± 0.4
Glucose (mg/100 mL)	85 ± 3	86 ± 3	89 ± 1	91 ± 1
Total cholesterol (mg/100 mL)	120 ± 6	110 ± 5	128 ± 12	91 ± 9^*^
Triglycerides (mg/100 mL)	19 ± 1	22 ± 3	22 ± 2	24 ± 4
Free fatty acids (mEq/L)	0.7 ± 0.1	1.1 ± 0.4	0.6 ± 0.1	1.2 ± 0.4
Total protein (g/100 mL)	6.2 ± 0.1	6.0 ± 0.1	6.2 ± 0.2	6.0 ± 0.3
Insulin (ng/mL)	0.29 ± 0.01	0.32 ± 0.02	0.36 ± 0.01	0.35 ± 0.02
Adiponectin (μg/mL)	24 ± 3	21 ± 2	21 ± 3	26 ± 4
AST (U/L)	32 ± 1	35 ± 2	33 ± 4	35 ± 4
ALT (U/L)	36 ± 3	35 ± 1	45 ± 3	53 ± 6

Values are presented as means ± standard error. ^*^Significantly different (*p* < 0.05) from the values at week 0 in the own group. ALT, alanine aminotransferase; AST, aspartate aminotransferase.

## 4 Discussion

Remarkable changes observed in the dogs fed diet supplemented with shiitake powder included hypocholesterolemia and increased sirtuin1 mRNA expression in circulating leukocytes. Eritadenine, a type of alkaloid and a hypocholesterolemic compound found in shiitake mushrooms, has a wide range of effects on lipid metabolism, such as increases in the liver microsomal phosphatidylethanolamine concentration, decreases in the liver microsomal Δ6-desaturase activity, and alterations in the composition of fatty acids and lipid molecular species profile in the liver and plasma (21). The hypocholesterolemic action of eritadenine may be associated with a modification in hepatic phospholipid metabolism, leading to decreased secretion of lipoprotein as a cholesterol transporter to the blood (21). On the other hand, excess doses of eritadenine have been reported to markedly increase the content of liver triglycerides and produce fatty liver (19). Although the dogs were supplemented with a massive amount of eritadenine in this study, their BW, BCS, plasma glucose, total protein, and triglyceride concentrations and the activities of hepatic deviation enzymes (ALT and AST) did not change, and symptoms of fatty liver were not observed. The increase in plasma FFA concentration and SOD activities may be induced by some components in shiitake powder that activate sirtuin1. Sirtuin is an NAD^+^-dependent protein deacetylase and a master metabolic regulator in different metabolic tissues (22). It depresses lipid synthesis and activates fatty acid oxidation, ameliorating ROS stress (22). Activated sirtuin1 has been reported to increase SOD activity (23, 24).

Sirtuin1 is a crucial regulator involved in white adipose tissue browning (25), and it facilitate erythropoietin production to enhance metabolic activity (26). Upregulation of sirtuin1 mRNA is linked to the downregulation of lipid synthesis (27) and the upregulation of glucose utilization, fatty acid oxidation, and glycolysis to prevent obesity and obesity related diseases (28). Polyphenols such as resveratrol have been shown to increase sirtuin1 activity (29, 30). Treatment with *Coriolus versicolor*, a mushroom species, has been shown to induce the effects of sirtuin1 and preparations have been applied to improve neurodegeneration (31). Our results suggest that shiitake mushrooms may also contain useful components to activate sirtuin1. Further studies are needed to clarify the components that activate sirtuin1 in the shiitake powder (see Table 3).

**Table 3:** Changes in leukocyte mRNA expression of sirtuin1 and plasma superoxide dismutase activity in the blood of dogs fed diet supplemented with shiitake powder.

	Control (*n* = 4)		Shiitake supplemented (*n* = 4)	
	Week 0	Week 4	Week 0	Week 4
Sirtuin1/β-actin	0.07 ± 0.02	0.13 ± 0.09	0.13 ± 0.06	0.24 ± 0.03^*^
SOD (units/mL)	22.7 ± 2.7	38.0 ± 13.8	20.2 ± 5.5	103.0 ± 12.4^*^

Values are presented as means ± standard error. ^*^Significantly different (*p* < 0.05) from the value at week 0 in the same group.

This study is preliminary and limitations include a small number of samples and biological and environmental variables (age and sex). The placebo group for shiitake powder was not set because the number of animals was not enough. The optimal dose of shiitake powder as a supplement to be administered to dogs was not examined. The microbiological contamination of dog food bowls was not examined in this study (32). In recent years, to understand the nutrition and health states in animals, the new learning methods like the flipped classroom and peer-assisted learning (33) and the usage of Instagram as a social media platform (34) are developed and applied in veterinary education. To judge the effectiveness of newly developed supplements on animal nutrition and health, these methods should be applied in the obese animals in the future. Further studies are needed in overweight and obese dogs to evaluate the usefulness of shiitake powder as an anti-obesity supplement.

In conclusion, shiitake powder was administered to healthy dogs at a dose of 800 mg/kg BW/day for 4 weeks. The dose corresponded to 0.60–0.65 mg/kg/day (likely to induce fatty acid accumulation) of eritadenine, a hypocholesterolemic factor. None of the dogs examined showed signs of disease during the experiment. Body weight and BCS did not change after shiitake supplementation. In the plasma of the dogs that received the supplement, total cholesterol levels decreased significantly and FFA concentrations increased. In the dogs administered the shiitake powder, leukocyte sirtuin1 mRNA expression and plasma SOD activities increased significantly. The shiitake powder increased antioxidative activity and may be a useful supplement to ameliorate age-related diseases, including obesity in dogs.

## Data availability statement

The original contributions presented in the study are included in the article/supplementary material, further inquiries can be directed to the corresponding author.

## Ethics statement

The animal study was approved by Narita Animal Science Laboratory Co., Ltd. Research Animal Ethical Committee. The study was conducted in accordance with the local legislation and institutional requirements.

## Author contributions

AK: Conceptualization, Data curation, Formal analysis, Investigation, Methodology, Resources, Writing – original draft. TA: Conceptualization, Project administration, Supervision, Validation, Writing – review & editing.
